# Determinants of fertility experience among reproductive women age (15–49) in Ethiopia: Application of count regression models

**DOI:** 10.1371/journal.pone.0312999

**Published:** 2024-10-31

**Authors:** Bantie Getnet Yirsaw, Birhan Ambachew Taye, Wullo Sisay Seretew, Aychew Kassa Belete, Tigabu Kidie Tesfie

**Affiliations:** 1 Department of Epidemiology and Biostatistics, Institute of Public Health, College of Medicine and Health Sciences, University of Gondar, Gondar, Ethiopia; 2 Department of Statistics, Woldia University, Woldia, Ethiopia; 3 Department of Sport Science, Woldia University, Woldia, Ethiopia; Haramaya University, ETHIOPIA

## Abstract

**Introduction:**

Fertility refers to the average number of children born to a woman over her reproductive years. Due to cultural norms, economic development, education, access to healthcare, and government policies, fertility rates vary significantly across different countries and regions of the world. Ethiopia is one of the developing countries in Sub-Sahara Africa and its fertility rate has consistently been one of the highest in the world. Hence the main goal of this study was to identify the leading factors for the total number of children born per mother in Ethiopia.

**Methods:**

This study used the most recent secondary data obtained from the 2019 Ethiopia Mini Demographic and Health Survey. A total weighted sample of 8885 women aged 15 to 49 was included in the study. The primary outcome variable in this study was the number of children born per mother, and different socio-demographic and economic factors that could influence fertility experience were included. The analysis was performed using STATA version 17 software. Negative Binomial regression model was used to assess the association between outcome and predictor variables. A p-value <0.05 was considered to be statistically significant.

**Results:**

From a total of 8885 women, on average approximately 2.73 children were born per mother. Age of mothers (IRR = 1.08, 95% CI: (1.077, 1.082)), religion for Muslim (IRR = 1.13, 95% CI: (1.08, 1.18)) and others (IRR = 1.16, 95% CI: (1.11, 1.22)), level of education secondary and above (IRR = .61, 95% CI: (.58, .65)) and primary (IRR = .84, 95% CI: (.81, .87)), household member of six to nine (IRR = 1.24, 95% CI: (1.20, 1.28)) and household members more than nine people (IRR = 1.14, 95% CI: (1.07, 1.21)), wealth index for rich (IRR = .94, 95%, CI: (.91, .98)), marital status for not married (IRR = .49, 95%, CI: (.48, .51)), pregnant mothers (IRR = 1.08, 95%, CI: (1.02, 1.13)) and contraceptive users (IRR = 1.12, 95%, CI: (1.09, 1.16)) were the signficant variables for number of children ever born per mother.

**Conclusion:**

In Ethiopia, the average number of children born per mother was approximately 2.73. Poisson and Negative Binomial regression models were compared, and we found the Negative Binomial regression model to be better to identify the determinants of fertility experience in Ethiopia. Age of mothers, religion, current pregnancy, use of contraceptive methods, mothers’ education level, household members, wealth index, number of children aged five and under, region (Somali, Gambela, and Addis Ababa), and marital status were the determinants responsible for fertility experience among mothers in Ethiopia. These findings are helpful for policymakers and healthcare professionals for developing targeted interventions and programs aimed at improving the high fertility rate experience in Ethiopia.

## Introduction

The ability of a person or a couple to conceive a child naturally is referred to as fertility and also defined as the average number of children born to a woman over her reproductive years [1]. It is a complicated process that includes the successful fertilisation and implantation of the embryo in the woman’s uterus, as well as the creation of healthy eggs by women and viable sperm by men [2].

It is also an essential aspect of human reproductive health and plays a crucial role in the continuity of the human population. Both men and women have unique factors that contribute to fertility [3].

Fertility rates vary significantly across different countries and regions of the world. According to [4], the global average fertility rate was around 2.4 children per woman. However, this average masks significant variations. Many developed countries, particularly in Europe and East Asia, have experienced declining fertility rates. Factors influencing declining fertility rates in developed countries include women’s increasing participation in the labor force, delayed marriages, changing societal norms, economic factors, and the availability of family planning services. However, it is important to note that fertility rates can fluctuate over time due to a combination of societal, economic, and policy changes [5].

Furthermore, these countries often have higher levels of education, better access to contraceptives, and advanced healthcare systems, which contribute to lower fertility rates. Some countries even have fertility rates below the replacement level of 2.1, which means their populations are shrinking without immigration [6].

In contrast, high fertility rates are generally observed in less developed regions with poorer socioeconomic conditions, limited healthcare access, and lower levels of education. For example, Sub-Saharan Africa has the highest fertility rates in the world, with countries like Niger, Somalia, and Chad having average fertility rates above 6 children per woman [7].

Moreover, in developing countries, several factors influence fertility rates, including cultural norms, economic development, education, access to healthcare, and government policies [8]. For instance, a study conducted in Nigeria using count regression models found that contraceptive use, residing with a partner, having more than one wife, age at marriage, women’s work status, currently breastfeeding, partners’ age, level of education (secondary and higher), wealth quintile, religion, ethnicity, and region were statistically significantly determinants associated with fertility [9].

Another study conducted in Ethiopia found place of residence as one of the significant factors for fertility. A mother who lived in a rural area has a variation with their counterparts. This variation could be access to education, media, and providing employment opportunities in the modern economic sector [10].

Ethiopia is one of the developing countries in Sub-Sahara Africa and its fertility rate has consistently been one of the highest in the world [11]. According to data from the United Nations, the total fertility rate in Ethiopia was estimated to be 4.2 children per woman in 2021, down from 4.8 in 2010. This rate is significantly higher than the global average of around 2.3 children per woman, and the significant variables that lead to the rate above the global level are the education level of mothers, income level, religious characteristics, limited health care infrastructure, and limited access to contraceptives [12].

The Ethiopian government has been implementing various programs and initiatives to address the issue of high fertility rates. These include improving access to healthcare services, expanding family planning programs, promoting girls’ education, and raising awareness about the benefits of smaller family sizes [13]. However, progress has been slow, and further efforts are needed to effectively reduce fertility rates in Ethiopia.

Therefore, the main focus of this research was to estimate the fertility rate and identify the factors for fertility in Ethiopia using Ethiopian Mini Demographic Health Survey Data, 2019 with an application of count regression models. Hence, identifying the significant predictors can help in predicting future fertility trends and planning resources accordingly. Furthermore, policy makers and health professionals can use this information to target interventions and programs aimed at improving reproductive health outcomes in the country.

## Data and methods

### Study design and setting

Secondary data obtained from the 2019 Ethiopia Mini Demographic and Health Survey (EMDHS, 2019) were used in this study. The survey collected demographic and health information from a nationally representative sample, and was conducted from March 21, 2019, to June 28, 2019. A complete list of 149,093 enumeration areas was created for the 2019 Ethiopian Population and Housing Census, and each enumeration area covered an average of 131 households.

Ethiopia was divided into nine geographical regions and two administrative cities, and each region was stratified into urban and rural areas, yielding 21 sampling strata. Samples of enumeration areas were selected independently from each stratum in two stages. Where enumeration areas were first-stage units, households were second-stage units, and required households were selected. In the selected households, women aged 15–49 were interviewed. A total of 8885 weighted samples of reproductive age (15–49) were taken. More explanations can be found in the 2019 EMDHS report [14].

### Study variables

#### Outcome variable

The response/outcome variable was the number of children born per mother.

#### Independent variables

The primary determinant variables for the number of children ever born per mother are level of education for mothers(0. no education, 1. Primary, 2. secondary and above), number of household members(1. five or less, 2. six up to nine, 3. greater than nine), age of mothers, currently pregnancy status of mothers(0. no, 1. yes), use of contraceptive methods(0. no, 1. yes), number of children aged five and under(0. no child, 1. one, 2. two, 3. more than two), marital status(1. married, 2. single/divorced/separated), place of residence(1. urban, 2. rural), religion(1. orthodox, 2. muslim, 3. others), wealth index(1. poor, 2. middle, 3. rich), and region(1. Tigray, 2. Afar, 3. Amhara, 4. Oromia, 5. Somali, 6. Benshangul Gumuz, 7. South Nation Nationalities and Peoples, 8. Gambela, 9. Harari, 10. Addis Ababa, 11. Dire Dawa) were discussed in this paper.

### Method of analysis

Count regression models are statistical models used to analyse data where the variable of interest represents a count/discrete like the total children ever born per mother and the independent variables are of any type. In our study, the outcome variable was expressed as discrete (number of children ever born per mother). The analysis was performed using STATA version 17 statistical software. Among the different count regression models, the Poisson regression model (for equip-dispersion), Negative Binomial regression model (for overdispersion), Hurdle regression model(to handle both overdispersion and underdispersion), Zero-inflated Poisson regression model (for excess zeros in Poisson regression), Zero-inflated Negative Binomial regression model (for excess zeros in Negative Binomial regression) were the appropriate models for our study.

#### Fitting countig regression models

*Poisson regression model*. This model is appropriate for count/discrete data when the mean and variance are equal.

The probability mass function for Poisson distribution is

$$p(y_{i})=\frac{e^{-\lambda_{i*}}\lambda_{i}^{y_{i}}}{y_{i}^{!}}$$ (1)

where *y*_*i*_ is the number of children born per mother and *λ_i_* is the average number of children born per mother for each *y*_*i*_

Then Poisson regression model with p explanatory variables say, *x*_1_,*x*_2_,…,*x*_*p*_ can be written as:

E(yixi)=λi=exp(xi'βi) (2)

$$\log(\lambda_{i})=\beta_{0}+\beta_{1}x_{i1}+\beta_{2}x_{i2}+\cdots +\beta_{p}x_{ip}$$ (3)

*Negative binomial regression model*. In modelling, Poisson regression model E(yixi)=λi=var(yixi) is true. But in many circumstances, this may not be true and we found another count regression model that tolerates this property, and the Negative Binomial regression model is appropriate in the presence of overdispersion in the data (i.e. E(yixi)>var(yixi)).

The general Negative Binomial distribution with the gamma mixture of poisson random variables can be written as:

f(yixi)=γ(yi+α−1)yi!γ(α−1)α−1α−1+λiα−1λiα−1+λiyi,yi=0,1,2,…andα>0 (4)

E(yixi)=λi=exp(xi'βi) (5)

var(yixi)=λi(1+αμi) (6)

Where *λ_i_* is the conditional mean and *λ_i_* (1+*αμ*_*i*_) is the conditional variance.

The model can be written as Eq (3) and *λ_i_* is the average number of children born per mother for each *y*_*i*_.

#### Model building and testing

For any model with many independent variables, one is usually concerned with the goal of selecting those variables that result in the best model within the scientific context of the problem [15].

For the model-building process, the following two steps are recommended: Firstly, the selection process should begin with a univariate analysis of each variable, and secondly, for the selection of variables, the multivariate analysis will follow based on the results of the univariate analysis along with all variables. Once a model has been developed using these two stages, the goodness-of-fit of the model should be checked using; the two common approaches, Pearson’s Chi-square statistic and the likelihood ratio statistic, which are based on the comparison of the fitted and the observed counts. The large values of Pearson’s Chi-square statistic and likelihood-ratio statistic indicate a lack of fit of the model [16].

### Ethical approval and consent

As the study used existing public survey data sets, which we requested from the MEASURE DHS program, permission was given to download data sets. Hence, ethical approval was not necessary for this study, and there are no names of individuals or household addresses in the data file.

### Results

Table 1 depicts that from a total of 8885 women, on average approximately 2.73 children are born per mother. The minimum number of children born per mother was zero, followed by a maximum of fifteen.

**Table 1:** Characteristics of study participants.

**Variables**	**Number of mothers (weighted)**		**Number of mothers (%)**	
**Place of Residence**				
Urban	2861		32.20	
Rural	6024		67.80	
**Religion**				
Orthodox	3685		41.47	
Muslim	2619		29.48	
Others	2581		29.05	
**Currently Pregnant**				
Yes	689		7.75	
No	8196		92.25	
**Mothers’ Education**				
No education	3589		40.40	
Primary	3701		41.65	
Secondary and above	1595		17.95	
**Number of children five and under**				
No child	3735		42.04	
One	3007		33.84	
Two	1741		19.59	
Three or more	402		4.53	
**Household Members**				
Five or less	4522		50.90	
Six up to nine	3833		43.13	
Greater than nine	530		5.97	
**Region**				
Tigray	629		7.08	
Afar	85		0.96	
Amhara	2026		22.80	
Oromia	3347		37.67	
Somali	420		4.73	
Benishangul Gumuz	98		1.11	
South Nations Nationalities and Peoples	1705		19.19	
Gambela	41		0.46	
Harari	27		0.30	
Addis Ababa	442		4.97	
Dire Dawa	65		0.73	
**Use of contraceptive methods**				
Yes	2556		28.76	
No	6329		71.24	
**Marital status**				
Married	5743		64.64	
Single/Divorced/Separated	3142		35.36	
**Wealth Index**				
**Poor**	3052		34.35	
Middle	1671		18.81	
Rich	4162		46.84	
**Summary of continuous variables**				
**Variables**	**Mean**	**Variance**	**Minimum**	**Maximum**
Age of mothers	27.56	84.82	15	49
Children ever born per mother	2.73	8.53	0	15

According to Table 1, the average age of mother respondents was around 27.56 years old, with a standard deviation of 9.21. For fertility, the lowest/minimum age of mothers was 15 years, and the highest age was 49 years.

Table 1 showed that the majority of the respondents, 6024 (67.80%) were from rural areas and about 2861 (32.20%) were from urban areas. Results also showed that respondents from the Oromia region had a maximum of 3347 (37.67%) followed by the Amhara 2026 (22.80%) and the lowest number of respondents, were 27 (0.30%) from the Harari region. Table 1 also showed that a greater proportion of respondents, 3685 (41.47%) of the participants were Orthodox than Muslim and other religions. About the majority 8196 (92.25%) of the participants were not pregnant during the interview or the study. About 3701 (41.65%) of the respondents have primary education level and about 3589 (40.40%) of the respondents have no formal education. Regarding the household members, the analysis suggested that approximately half of the proportion 4522 (50.90%) were five or less; and the majority of the respondents 3735 (42.04%) were had no child five and under. For the usage of contraceptive methods, the result reported that, 6329 (71.24%) of the respondents did not use contraceptive methods, and the highest proportion of the participants, 5743 (64.64%) were married. In addition, the largest proportion of respondents were from high-income levels (rich) 4162 (46.84%).

#### Model building

As the result from the above Table 2 showed, the variance of the total children ever born per mother was approximately four times the mean, which is an indication of overdispersion. Even though the result suggests the presence of overdispersion in the data, we do not verify using only the outcome variable. Therefore, we should test the presence of overdispersion outcome variable conditional on the explanatory variables.

**Table 2:** Test of overdispersion conditional on independent variables.

Total children ever born	Coefficient	Standard error	T	p-value	95% confidence interval
**Uhat**	.02	.01	4.40	0.0000	[.01, .03]

Overdispersion test (H0: equidispersion)

The result (Table 2) p-value indicates the presence of significant overdispersion in the dependent variable conditional on explanatory variables. Hence, in both cases, the poisson regression model is not appropriate for our data, and this suggests Negative Binomial regression model is appropriate.

Among the list of explanatory variables (Table 3) depicted the age of mothers, religion, mothers’ education level, household members, wealth index, children whose age five and under, region (Somali, Gambela, Addis-Ababa), use of contraceptive methods, pregnancy status, place of residence, and marital status were statistically significant determinants for fertility experience.

**Table 3:** Estimated values of the coefficients, Incidence Rate Ratio (IRR), 95% confidence intervals (95% CI.), and p-values (P>|z|) of the explanatory variables for both multivariable and bivariable analysis.

Variables		Multi-variable Analysis			Bivariable Analysis	
		Coefficient (95% CI.)	IRR (95% CI.))	P>|z|	IRR (95% CI.))	P>|z|
**Age of mothers**		.08(.07, .08)	1.08(1.077, 1.082)	0.000	1.10(1.096, 1.101)	0.000
**Region**	**Tigray**	**reference category**				
	Afar	.02(-.06, .10)	1.02(.94, 1.10)	0.665	1.23(1.08, 1.39)	0.001
	Amhara	-.01(-.08, .05)	.99(.93, 1.05)	0.717	1.06(.94, 1.18)	0.366
	Oromia	.06(-.01, .13)	1.07(.99 1.14)	0.072	1.23(1.10, 1.38)	0.000
	Somali	.10(.02, .18)	1.10(1.02, 1.19)	0.018	1.39(1.23, 1.58)	0.000
	Benishangul	-.001(-.07, .07)	.99(.93, 1.07)	0.975	1.17(1.03, 1.32)	0.011
	South Nation Nationalities	.03(-.04, .10)	1.03(.96, 1.10)	0.456	1.26(1.13, 1.41)	0.000
	Gambela	.18(.10, .26)	1.19(1.11, 1.29)	0.000	1.11(.98, 1.25)	0.110
	Harari	.04(-.04, .12)	1.04(.96, 1.13)	0.336	.87(.77, .99)	0.029
	Addis Ababa	-.27(-.37, -.17)	.76(.69, .84)	0.000	.39(.35, .45)	0.000
	Dire Dawa	-.06(-.14, .02)	.94(.87, 1.02)	0.165	.82(.72, .92)	0.001
**Place of residence**	**Urban**	**reference category**				
	Rural	.09(.04, .13)	1.09(1.05, 1.14)	0.000	1.83(1.73, 1.93)	0.000
**Religion**	**Orthodox**	**reference category**				
	Muslim	.12(.07, .16)	1.13(1.08, 1.18)	0.000	1.42(1.34, 1.50)	0.000
	Others	.15(.10, .20)	1.16(1.11, 1.22)	0.000	1.37(1.28, 1.47)	0.000
**Mothers education level**	**No education**	**reference category**				
	Primary	-.18(-.21, -.14)	.84(.81, .87)	0.000	.39(.37, .41)	0.000
	Secondary and above	-.49(-.55, -.43)	.61(.58, .65)	0.000	.23(.21, .24)	0.000
**Household members**	**Five or less**	**reference category**				
	Six up to nine	.21(.18, .25)	1.24(1.20, 1.28)	0.000	2.03(1.93, 2.14)	0.000
	More than 9	.13(.07, .19)	1.14(1.07, 1.21)	0.000	1.96(1.77, 2.16)	0.000
**Wealth index**	**Poor**	**reference category**				
	Middle	.003(-.04, .04)	1.00(.96, 1.05)	0.888	.91(.84, .98)	0.012
	Rich	-.06(-.10, -.02)	.94(.91, .98)	0.003	.56(.53, .59)	0.000
**Currently Pregnant**	**No**	**reference category**				
	Yes	.07(.02, .12)	1.08(1.02, 1.13)	0.004	1.02(.93, 1.12)	0.000
**Contraceptive use**	**No**	**reference category**				
	Yes	.12(.08, .15)	1.12(1.09, 1.16)	0.000	1.21(1.15, 1.29)	0.000
**Children five and under**	No child	**reference category**				
	One	.30(.26, .34)	1.35(1.30, 1.40)	0.000	1.44(1.35, 1.53)	0.000
	Two	.57(.52, .61)	1.77(1.69, 1.84)	0.000	1.91(1.78, 2.04)	0.000
	More than two	.69(.63, .75)	1.99(1.88, 2.12)	0.000	2.34(2.11, 2.60)	0.000
**Marital Status**	**Married**	**reference category**				
	Single/divorced/separated	-.72(-.76, -.67)	.49(.47, .51)	0.000	.22(.21, .23)	0.000
**Constant**		-1.76(-1.88, 1.64)	.17(.15, .19)	0.000		
**/alpha**		-3.70				
**Alpha**		.025				

**Note:** **/alpha** –This is the estimate of the log of the dispersion parameter, **alpha**

**alpha** –This is the estimate of the dispersion parameter. if alpha = 0, then the model will be Poisson and if **alpha >** 0, then the model will be Negative Binomial.

The coefficient for the age of mothers indicates that as the mother’s age increases by one year, the difference in the logs of odds expected number of children born per mother would be expected to increase by .08 unit (95% CI: (.07, .08)), or as the mothers age increases by one year the rate of having children would be expected to increase by a factor of 1.08 (IRR = 1.08, 95% CI: (1.077, 1.082)), keeping other variables constant.

According to results from the region, mothers from the Somali region (IRR = 1.10, 95% CI: (1.02, 1.19)) and from the Gambela region (IRR = 1.20, 95%, CI: (1.11, 1.29)) more likely to have children, whereas mothers from Addis-Ababa town administration (IRR = .76, 95% CI: (.69, .84)) as compared to mothers from the Tigray region.

Mothers whose religion is Muslim (IRR = 1.12, 95% CI: (1.07, 1.18)) and others (IRR = 1.16, 95% CI: (1.11, 1.22)) were more likely to have children as compared to mothers whose religion were Orthodox.

The result also showed that mothers’ educational level and the number of children ever born per mother are inversely associated. Mothers whose education level was secondary and above (IRR = .61, 95% CI: (.58, .65)) were 39% less likely, and mothers whose education level was primary (IRR = .84, 95% CI: (.81, .87)) were 16% less likely to bear children than mothers whose education level were no education.

The incidence rate ratio presented among those who are living in a household with household members of six up to nine people (IRR = 1.24, 95% CI: (1.20, 1.28)) and household members more than nine people (IRR = 1.14, 95% CI: (1.07, 1.21)) were more likely to bear children than those living in a household with a family member of five or fewer respectively.

The result (Table 3) also reported that mothers from rural areas (IRR = 1.09, 95% CI: (1.05, 1.14)) were more likely to bear children than mothers who were living in urban areas.

Level of income (wealth index) was significantly associated with children ever born, rich mothers (IRR = .94, 95%, CI: (.91, .98)) 6% less likely to bear children than mothers who were poor.

The pregnancy status of mothers was also associated with the number of children ever born, mothers who were pregnant (IRR = 1.08, 95%, CI: (1.02, 1.13)) were more likely to bear children than mothers who were not pregnant. Similarly, mothers who used contraceptive methods (IRR = 1.12, 95%, CI: (1.09, 1.16)) had less risk of fertility.

The result also revealed that marital status and the number of children ever born were negatively associated, unmarried mothers were (IRR = .49, 95%, CI: (.47, .51)) 51% less likely to bear children as compared to mothers who were married. On the other hand, mothers who have children five and under those who have one child (IRR = 1.35, 95%, CI: (1.30, 1.40)), two children (IRR = 1.76, 95%, CI: (1.69, 1.84)) and more than two children (IRR = 1.99, 95%, CI: (1.87, 2.12)) were more likely to have high fertility than mothers who have no child respectively.

Additionally, results (Table 3) can also be interpreted using the Poisson regression coefficients. Hence, regarding the age of mothers, keeping other variables constant, if mothers age increase by one year, the difference in logs of the expected number of children born per mother increases by 0.08.

With regard to place of residence, the difference in the logs of the expected number of children born per mother is expected to be 0.09 units higher for mothers who are living in rural areas than urban, holding constant other independent variables. Similar interpretations can be performed for the rest of the categorical variables.

## Discussion

This study analyses the determinant of fertility experience among reproductive women age using the Ethiopian Mini Demographic Health Survey, 2019 in Ethiopia, and revealed that on average 2.73 children are born per mother. Our study showed, age of mothers, religion, pregnancy status, use of contraceptive methods, mothers’ education level, size of household members, wealth index, number of children who were aged five and under, region (Somali, Gambela, and Addis Ababa), and marital status were significant determinants of fertility experience.

The findings of this study showed that mothers’ age was a significant determinant of fertility experience. As the mother’s age increases by one year, the rate of childbearing would be expected to increase. This may be due to the fact that as the mother’s age increases, the exposure to marriage increases. This is in agreement with a study conducted in Ethiopia that shows the increase in fertility experience with increasing age [17].

In this study, we found a significant association between religion and the number of children ever born. Mothers whose religion was Muslim and others were more likely to have children as compared to mothers whose religion was Orthodox. This might be due to some cultures and belief systems, having many children is seen as a sign of blessing and is encouraged by religious teachings. This finding is in line with the study conducted in Ethiopia [18].

Regarding a mother’s education level, educated mothers are less likely to bear children than non-educated mothers. The findings also confirm results from other studies [9, 17]. This may be due to educated mothers having greater access to contraception, having more awareness about family planning, and financial costs associated with raising a child, knowing about advantages of birth interval or spacing, and they spending their time at school.

On the other hand, the result showed that mothers who have six or more family members were more likely to bear children than their counterparts. The possible explanations might be that larger families may have more resources and support available to help care for children, making it easier for mothers to have more children. This finding is agreed with a study conducted in Ethiopia [18]. Similary, the result also revealed that the number of children aged five and under was another significant determinant of fertility experience. Mothers who have two or more children had a lower risk of bearing children than mothers who have one or fewer children. One possible explanation may that to mothers who have already had children may have more experience in pregnancy and childbirth, which could lead to better prenatal care and healthier outcomes for subsequent pregnancies that promote the mother to have more children.

The level of income and marital status of mothers were significant determinants for the number of children ever born per mother. Mothers who have high (rich) and middle-income levels were less likely to bear children than mothers who were poor. This may be due the fact that mothers from higher-income households may have greater access to family planning resources, education, and empowerment, leading them to make more informed decisions about the number of children to bear. Similarly, unmarried (single, divorced, separated) mothers were less likely to have children as compared to married mothers. Different reasons might be possible explanations, such as lack of emotional and practical support in unmarried (single, divorced, separated) mothers that married mothers often have from their husbands. In addition, unmarried mothers may have to juggle multiple responsibilities such as work, childcare, and household chores on their own. Besides, unmarried mothers may have experienced difficulties in their previous relationships or may be dealing with the emotional aftermath of a divorce or separation, which could make them hesitate to have more children. These explanations is supported by previous finding [9].

Place of residence was another significant determinant variable in this study; mothers who were living in rural areas were more likely to bear children than mothers who were living in urban areas [10]. This may be due to the proportion of educated people live in urban areas than rural areas, low conraceptive knowledge and early marriage for mothers who are living in rural areas. This can lead in higher fertility rates and a greater likelihood of women in rural areas becoming pregnant and bearing children.

## Strength and limitation

### Strength

The study examines a wide range of predictors, providing a comprehensive understanding of fertility experience in Ethiopia. The use of recent data from a national survey helps ensure the relevance and applicability of the findings to the current context in Ethiopia at the national level.

### Limitations

This study is based on data from the 2019 Ethiopian Mini Demographic Health Survey, which may not capture all relevant factors influencing fertility experience. There may be other unmeasured variables that could also influence fertility experience among women in Ethiopia.

## Conclusion

This study demonstrated the average number of children born per mother was approximately 2.73. The study also compared two models (Poisson and Negative Binomial regression models) and found the Negative Binomial regression model to be the best to identify the determinants of the number of children ever born per mother. Age of mothers, religion, current pregnancy, use of contraceptive methods, mothers’ education level, household members, wealth index, number of children aged five and under, region (Somali, Gambela, and Addis Ababa), and marital status were the determinants responsible for fertility experience among mothers in Ethiopia. These findings are helpful for policymakers and healthcare professionals for developing targeted interventions and programs aimed at improving the high fertility rate experience in Ethiopia.

The researchers suggest that further studies can be considered as extensions and improvements to the Negative Binomial regression model, such as multilevel count regression models that incorporates higher levels of the nesting hierarchy.
